# The mortality burden of frailty in hip fracture patients: a nationwide retrospective study of cause-specific mortality

**DOI:** 10.1007/s00068-022-02204-6

**Published:** 2022-12-26

**Authors:** Maximilian Peter Forssten, Ahmad Mohammad Ismail, Ioannis Ioannidis, Per Wretenberg, Tomas Borg, Yang Cao, Marcelo A. F. Ribeiro, Shahin Mohseni

**Affiliations:** 1 Department of Orthopedic Surgery, Orebro University Hospital, 701 85 Orebro, Sweden; 2 Division of Trauma and Emergency Surgery, Department of Surgery, Orebro University Hospital, 701 85 Orebro, Sweden; 3 School of Medical Sciences, Orebro University, 702 81 Orebro, Sweden; 4 Clinical Epidemiology and Biostatistics, School of Medical Sciences, Orebro University, 701 82 Orebro, Sweden; 5 Pontifical Catholic University of São Paulo, São Paulo, Brazil; 6 Trauma, Burns, Critical Care and Acute Care Surgery, Department of Surgery, Sheikh Shakhbout Medical City-Mayo Clinic, Abu Dhabi, United Arab Emirates

**Keywords:** Frailty, Hip fracture, Postoperative mortality, Cause-specific mortality, Poisson regression

## Abstract

**Purpose:**

Frailty is a condition characterized by a reduced ability to adapt to external stressors because of a reduced physiologic reserve, which contributes to the high risk of postoperative mortality in hip fracture patients. This study aims to investigate how frailty is associated with the specific causes of mortality in hip fracture patients.

**Methods:**

All adult patients in Sweden who suffered a traumatic hip fracture and underwent surgery between 2008 and 2017 were eligible for inclusion. The Orthopedic Hip Frailty Score (OFS) was used to classify patients as non-frail (OFS 0), pre-frail (OFS 1), and frail (OFS ≥ 2). The association between the degree of frailty and both all-cause and cause-specific mortality was determined using Poisson regression models with robust standard errors and presented using incidence rate ratios (IRRs) with corresponding 95% confidence intervals (CIs), adjusted for potential sources of confounding.

**Results:**

After applying the inclusion and exclusion criteria, 127,305 patients remained for further analysis. 23.9% of patients were non-frail, 27.7% were pre-frail, and 48.3% were frail. Frail patients exhibited a 4 times as high risk of all-cause mortality 30 days [adj. IRR (95% CI): 3.80 (3.36–4.30), *p* < 0.001] and 90 days postoperatively [adj. IRR (95% CI): 3.88 (3.56–4.23), *p* < 0.001] as non-frail patients. Of the primary causes of 30-day mortality, frailty was associated with a tripling in the risk of cardiovascular [adj. IRR (95% CI): 3.24 (2.64–3.99), *p* < 0.001] and respiratory mortality [adj. IRR (95% CI): 2.60 (1.96–3.45), *p* < 0.001] as well as a five-fold increase in the risk of multiorgan failure [adj. IRR (95% CI): 4.99 (3.95–6.32), *p* < 0.001].

**Conclusion:**

Frailty is associated with a significantly increased risk of all-cause and cause-specific mortality at 30 and 90 days postoperatively. Across both timepoints, cardiovascular and respiratory events along with multiorgan failure were the most prevalent causes of mortality.

## Introduction

Frailty is a condition characterized by a reduced ability to adapt to external stressors because of a reduced physiologic reserve pursuant to the degeneration of multiple organ systems. As a result, frail patients are at a disproportionate risk of morbidity, disability and mortality [1–4]. While frailty increases with advancing age, it is not synonymous with old age, but rather an independent condition which has been found to be superior to a patient’s chronological age in predicting worse outcomes after surgery [5–7]. Frailty is particularly prevalent among hip fracture patients and may explain a substantial portion of the excess mortality observed in this patient population [8–12]. Subsequently, frailty appears to be a useful means of stratifying hip fracture patients. Using frailty, each patient’s risk of mortality may be estimated in ordered to identify high risk individuals with a disproportionate risk of postoperative mortality [13].

The ability to identify high risk patients will become increasingly important. Both the incidence of hip fractures as well as conditions associated with frailty such as dementia are projected to increase markedly as the average age of the human population continues to increase [14–17]. Simultaneously, hospital resources are predicted to become progressively strained over the same time period [18–21]. This will necessitate new, quick and efficient methods for allocating resources to those with the greatest need or restrictions in non-futile care. As part of this process, a better understanding is required of the most vulnerable patients, such as those that are frail. Previous investigations have studied the relationship between frailty and postoperative mortality after hip fracture surgery [8–12]. However, with the aid of Orthopedic Hip Frailty Score (OFS), this is the first study that aims to investigate how frailty is associated with cause-specific mortality in hip fracture patients.

## Methods

This study included all adult patients who underwent hip fracture surgery between 2008 and 2017, who had been registered in the Swedish National Quality Registry for Hip Fracture Patients, Rikshoft [22]. In order to reduce heterogeneity in the study population, pathological fractures, reoperations, and conservatively managed patients were excluded. Patients were also excluded if the data required for calculating the OFS was missing. The Swedish National Quality Registry for Hip Fracture Patients was queried for variables pertaining to the demographic, clinical, and operative characteristics of each patient; this included factors such as age, sex, American Society of Anesthesiologist (ASA) classification, type of fracture and type of surgery. By cross-referencing patients' Swedish social security number, comorbidity and morbidity data were acquired from the Swedish National Board of Health and Welfare Patient and Cause of Death registers and combined with the original dataset. This study was performed with the approval of the Swedish Ethical Review Authority (reference 2022-03107-02) in accordance with both the STROBE guidelines and the Declaration of Helsinki [23].

### Calculating the Orthopedic Hip Frailty Score

The OFS, a newly validated score for measuring frailty in hip fracture patients, was calculated based on the following variables: the presence of congestive heart failure, a history of malignancy (local or metastatic, excluding non-invasive skin cancer), institutionalization, non-independent functional status (i.e., requiring assistance with activities of daily life), and an age ≥ 85. A patient received 1 point for each variable, with the maximum possible score being 5. According to the original study, patients with an OFS ≥ 2 were considered frail [13].

### Statistical analysis

Patients were divided into three cohorts based on their OFS: non-frail (OFS 0), pre-frail (OFS 1), and frail (OFS ≥ 2). Demographics, clinical characteristics, and outcomes were summarized using a median and interquartile range (IQR) for non-normally distributed continuous variables and counts and percentages for categorical variables. The statistical significance of differences between these cohorts was evaluated using the Kruskal–Wallis test, or Chi-squared/Fisher's exact test, whichever was appropriate. The primary outcome was all-cause mortality, while secondary outcomes consisted of cause-specific mortality (cardiovascular, respiratory, cerebrovascular, sepsis, multiorgan failure, unknown). Mortality was evaluated after 30 days and 90 days postoperatively.

The association between frailty and 30 days as well as 90 days mortality (all-cause and cause-specific) was evaluated using Poisson regression models with robust standard errors. These models were adjusted for age, sex, ASA classification, type of fracture, type of surgery, myocardial infarction, cerebrovascular disease, peripheral vascular disease, diabetes mellitus, chronic kidney disease, chronic obstructive pulmonary disease, connective tissue disease, and liver disease. Results for these analyses are presented as incidence rate ratios (IRRs) with corresponding 95% confidence intervals (CIs).

Multiple imputation by chained equations was used to handle missing data; logistic regression was used for binary variables, Bayesian polytomous regression was used for nominal variables, and proportional odds models were used for ordinal variables. Analyses were performed using the tidyverse, haven, parallel, mice, robustbase, writexl, and rlist packages in the statistical software R 4.1.3 (R Foundation for Statistical Computing, Vienna, Austria) [24]. In all analyses, a two-sided *p* value less than 0.05 was considered statistically significant.

## Results

**Fig. 1:**
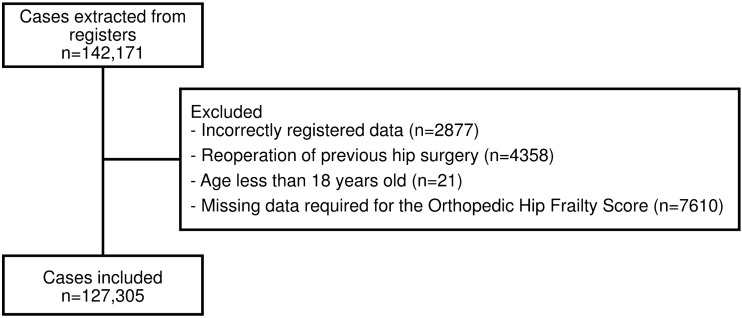
Flow chart describing selection of cohorts

After applying the inclusion and exclusion criteria 127,305 patients remained for further analysis (Fig. 1). 23.9% of patients (*N* = 30,463) were non-frail, 27.7% (*N* = 35,312) were pre-frail, while 48.3% (*N* = 61,530) had an OFS ≥ 2 and were therefore considered frail [13]. Compared to non-frail patients, pre-frail and frail patients were on average older (82 years and 88 years vs 75 years, *p* < 0.001), slightly more often female (67.6% and 69.6% vs 65.4% *p* < 0.001), and less fit for surgery according to their ASA classification (ASA ≥ 3: 54.5% and 71.0% vs 44.5%, *p* < 0.001). Pre-frail and frail were significantly more likely to undergo a hemiarthroplasty (27.9% and 29.2% vs 15.5%, *p* < 0.001) than a total hip replacement (7.7% and 2.4% vs 17.9%, *p* < 0.001) compared to non-frail patients. The majority of frail patients were older than 85 (76.2%) and had a non-independent functional status (85.4%). All comorbidities, except for liver disease and connective tissue diseases, were significantly more prevalent among frail patients. (Table 1).

**Table 1:** Demographics and clinical characteristics of non-frail, pre-frail, and frail hip fracture patients

	Non-frail patients (*N* = 30,463)	Pre-frail patients (*N* = 35,312)	Frail patients (*N* = 61,530)	*p* value
Age, median [IQR]	75 [68–80]	82 [76–87]	88 [85–91]	< 0.001
Sex, *n* (%)				
Female	19,915 (65.4)	23,863 (67.6)	42,825 (69.6)	< 0.001
Male	10,543 (34.6)	11,446 (32.4)	18,702 (30.4)	
Missing	5 (0.0)	3 (0.0)	3 (0.0)	
ASA classification, *n* (%)				
1	4225 (13.9)	1341 (3.8)	927 (1.5)	< 0.001
2	15,791 (51.8)	14,267 (40.4)	16,168 (26.3)	
3	9275 (30.4)	17,068 (48.3)	36,543 (59.4)	
4	675 (2.2)	2,143 (6.1)	7,058 (11.5)	
5	11 (0.0)	23 (0.1)	89 (0.1)	
Missing	486 (1.6)	470 (1.3)	745 (1.2)	
Type of fracture, *n* (%)				
Non-displaced cervical (garden 1–2)	4897 (16.1)	4582 (13.0)	7418 (12.1)	< 0.001
Displaced cervical (garden 3–4)	12,048 (39.5)	13,625 (38.6)	21,766 (35.4)	
Basicervical	945 (3.1)	1101 (3.1)	2225 (3.6)	
Peritrochanteric (two fragments)	5363 (17.6)	7008 (19.8)	13,327 (21.7)	
Peritrochanteric (multiple fragments)	4546 (14.9)	6198 (17.6)	11,925 (19.4)	
Subtrochanteric	2652 (8.7)	2782 (7.9)	4848 (7.9)	
Missing	12 (0.0)	16 (0.0)	21 (0.0)	
Type of surgery, *n* (%)				
Pins or screws	6764 (22.2)	5619 (15.9)	9692 (15.8)	< 0.001
Screws or pins with sideplate	7093 (23.3)	9188 (26.0)	17,170 (27.9)	
Intramedullary nail	6435 (21.1)	7900 (22.4)	15,214 (24.7)	
Hemiarthroplasty	4710 (15.5)	9869 (27.9)	17,974 (29.2)	
Total hip replacement	5446 (17.9)	2727 (7.7)	1451 (2.4)	
Missing	15 (0.0)	9 (0.0)	29 (0.0)	
OFS, *n* (%)				
0	30,463 (100.0)	0 (0.0)	0 (0.0)	< 0.001
1	0 (0.0)	35,312 (100.0)	0 (0.0)	
2	0 (0.0)	0 (0.0)	33,798 (54.9)	
3	0 (0.0)	0 (0.0)	21,859 (35.5)	
4	0 (0.0)	0 (0.0)	5428 (8.8)	
5	0 (0.0)	0 (0.0)	445 (0.7)	
Congestive heart failure, *n* (%)	0 (0.0)	2496 (7.1)	17,158 (27.9)	< 0.001
History of malignancy, *n* (%)	0 (0.0)	4391 (12.4)	12,215 (19.9)	< 0.001
Institutionalized, *n* (%)	0 (0.0)	2,700 (7.6)	28,280 (46.0)	< 0.001
Non-independent functional status, *n* (%)	0 (0.0)	13,343 (37.8)	52,574 (85.4)	< 0.001
Age ≥ 85, *n* (%)	0 (0.0)	12,382 (35.1)	46,883 (76.2)	< 0.001
Myocardial infarction, *n* (%)	768 (2.5)	1,651 (4.7)	5161 (8.4)	< 0.001
Cerebrovascular disease, *n* (%)	3017 (9.9)	6021 (17.1)	12,975 (21.1)	< 0.001
Peripheral vascular disease, *n* (%)	898 (2.9)	1627 (4.6)	3020 (4.9)	< 0.001
Diabetes, *n* (%)	3939 (12.9)	5422 (15.4)	9460 (15.4)	< 0.001
Chronic kidney disease, *n* (%)	688 (2.3)	1495 (4.2)	4318 (7.0)	< 0.001
COPD, *n* (%)	3019 (9.9)	4216 (11.9)	7398 (12.0)	< 0.001
Connective tissue disease, *n* (%)	1353 (4.4)	1795 (5.1)	3012 (4.9)	< 0.001
Liver disease, *n* (%)	424 (1.4)	479 (1.4)	408 (0.7)	< 0.001
Local cancer, *n* (%)	0 (0.0)	3518 (10.0)	10,287 (16.7)	< 0.001
Metastatic cancer, *n* (%)	0 (0.0)	873 (2.5)	1,928 (3.1)	< 0.001

Non-frail, pre-frail, and frail are defined as OFS 0, OFS 1, and OFS ≥ 2

*ASA* American Society of Anesthesiologists, *OFS* Orthopedic Hip Frailty Score, *COPD* chronic obstructive pulmonary disease

Crude all-cause mortality was also several times higher among pre-frail and frail patients at 30 days (4.2% and 12.1% vs 1.2%, *p* < 0.001) and 90 days (8.1% and 20.8% vs 2.5%) postoperatively, compared to non-frail patients. Across both time points, the primary causes of mortality were cardiovascular events, respiratory events, or multiorgan failure. However, pre-frail and frail patients exhibited higher rates of all specific causes of mortality (Table 2).

**Table 2:** Crude mortality rates in non-frail, pre-frail, and frail hip fracture patients

	Non-frail patients (*N* = 30,463)	Pre-frail patients (*N* = 35,312)	Frail patients (*N* = 61,530)	*p* value
30 days mortality, *n* (%)	380 (1.2)	1,500 (4.2)	7427 (12.1)	< 0.001
Cardiovascular, *n* (%)	156 (0.5)	557 (1.6)	3099 (5.0)	< 0.001
Respiratory, *n* (%)	87 (0.3)	304 (0.9)	1163 (1.9)	< 0.001
Cerebrovascular, *n* (%)	9 (0.0)	33 (0.1)	103 (0.2)	< 0.001
Sepsis, *n* (%)	8 (0.0)	33 (0.1)	137 (0.2)	< 0.001
Multiorgan failure, *n* (%)	109 (0.4)	522 (1.5)	2636 (4.3)	< 0.001
Unknown, *n* (%)	11 (0.0)	51 (0.1)	289 (0.5)	< 0.001
90 days mortality, *n* (%)	751 (2.5)	2858 (8.1)	12,802 (20.8)	< 0.001
Cardiovascular, *n* (%)	289 (0.9)	977 (2.8)	5015 (8.2)	< 0.001
Respiratory, *n* (%)	149 (0.5)	465 (1.3)	1708 (2.8)	< 0.001
Cerebrovascular, *n* (%)	20 (0.1)	88 (0.2)	276 (0.4)	< 0.001
Sepsis, *n* (%)	18 (0.1)	101 (0.3)	295 (0.5)	< 0.001
Multiorgan failure, *n* (%)	242 (0.8)	1,103 (3.1)	4814 (7.8)	< 0.001
Unknown, *n* (%)	33 (0.1)	124 (0.4)	694 (1.1)	< 0.001

Non-frail, pre-frail, and frail are defined as OFS 0, OFS 1, and OFS ≥ 2

This pattern remained consistent after adjusting for potential confounders. Frail patients exhibited a 4 times as high risk of all-cause mortality 30 days [adj. IRR (95% CI): 3.80 (3.36–4.30), *p* < 0.001] and 90 days postoperatively [adj. IRR (95% CI): 3.88 (3.56–4.23), *p* < 0.001] as non-frail patients. Of the primary cause of 30 days mortality, frailty was associated with a tripling in the risk of cardiovascular [adj. IRR (95% CI): 3.24 (2.64–3.99), *p* < 0.001] and respiratory mortality [adj. IRR (95% CI): 2.60 (1.96–3.45), *p* < 0.001] as well as a fivefold increase in the risk of multiorgan failure [adj. IRR (95% CI): 4.99 (3.95–6.32), *p* < 0.001] (Table 3).

**Table 3:** Incident rate ratios for postoperative mortality after hip fracture surgery

Mortality cause*	30 days mortality IRR (95% CI)	*p* value	90 days mortality IRR (95% CI)	*p* value
All-cause				
Non-frail	Reference		Reference	
Pre-frail	1.90 (1.67–2.16)	< 0.001	2.05 (1.87–2.24)	< 0.001
Frail	3.80 (3.36–4.30)	< 0.001	3.88 (3.56–4.23)	< 0.001
Cardiovascular				
Non-frail	Reference		Reference	
Pre-frail	1.61 (1.29–2.00)	< 0.001	1.62 (1.38–1.89)	< 0.001
Frail	3.24 (2.64–3.99)	< 0.001	3.08 (2.65–3.57)	< 0.001
Respiratory				
Non-frail	Reference		Reference	
Pre-frail	1.69 (1.27–2.26)	< 0.001	1.68 (1.35–2.10)	< 0.001
Frail	2.60 (1.96–3.45)	< 0.001	2.54 (2.04–3.16)	< 0.001
Cerebrovascular				
Non-frail	Reference		Reference	
Pre-frail	2.64 (1.05–6.62)	0.038	3.72 (2.03–6.82)	< 0.001
Frail	4.75 (1.88–11.98)	0.001	6.38 (3.46–11.77)	< 0.001
Sepsis				
Non-frail	Reference		Reference	
Pre-frail	2.22 (0.79–6.23)	0.129	3.30 (1.73–6.32)	< 0.001
Frail	3.79 (1.38–10.42)	0.010	4.14 (2.15–7.95)	< 0.001
Multiorgan failure				
Non-frail	Reference		Reference	
Pre-frail	2.36 (1.85–3.01)	< 0.001	2.54 (2.17–2.99)	< 0.001
Frail	4.99 (3.95–6.32)	< 0.001	4.86 (4.16–5.68)	< 0.001
Unknown				
Non-frail	Reference		Reference	
Pre-frail	2.50 (1.15- 5.42)	0.020	2.16 (1.36–3.43)	0.001
Frail	6.50 (3.09–13.67)	< 0.001	5.46 (3.50–8.51)	< 0.001

IRRs are calculated using Poisson regression models with robust standard errors. Missing values were managed using multiple imputation by chained equations. All analyses were adjusted for age, sex, American Society of Anesthesiologists classification, type of fracture, type of surgery, myocardial infarction, cerebrovascular disease, peripheral vascular disease, diabetes mellitus, chronic kidney disease, chronic obstructive pulmonary disease, connective tissue disease, and liver disease

*IRR* incident rate ratio,* CI* Confidence Interval

*Non-frail, pre-frail, and frail are defined as OFS 0, OFS 1, and OFS ≥ 2

## Discussion

This study is the largest to date studying the association between frailty and mortality in hip fracture patients and the first that investigates how frailty affects the risk of cause-specific mortality in hip fracture patients. As in the non-frail hip fracture population, the most common causes of mortality were attributable to cardiovascular events, respiratory events, and multiorgan failure. However, the regression analyses demonstrated that pre-frailty was associated with an approximate doubling of the risk of all-cause and cause-specific mortality, while frailty at least tripled the risk of all-cause as well as cardiovascular, respiratory, cerebrovascular, sepsis-related, and multiorgan failure-related mortality.

The role of frailty in hip fracture patients is an important and frequently studied question within orthopedics [8–12, 25]. In the current investigation approximately 48.3% of patients were classified as frail, which is consistent with at previous investigation by van de Ree et al.; however, the exact proportion will undoubtedly vary depending on how frailty is measured [11, 12, 25]. Several studies and systematic reviews have also reported an association between frailty and postoperative mortality in hip fracture patients [8–12]. The results of the current analysis were consistent with the estimates found by both Kwak et al. in their recent publication based on the National Inpatient Sample [Odds Ratio (95% CI), moderate frailty: 2.94 (1.91–4.51), high frailty: 5.99 (3.79–9.47)] as well as the meta-analysis by Ma et al. [Relative Risk (95% CI), 2.85 (1.67–4.85)] [9, 12]. The specific causes of death are also reflected in previous research with cardiovascular and respiratory events being among the most common in both the current analysis and prior investigations [26, 27].

Of note, while crude rates of mortality are several times higher in frail compared to non-frail patients, the distribution of the specific causes of mortality appear to be relatively similar. In both cohorts, patients are most likely to die of cardiovascular and respiratory events as well as multiorgan failure. This raises the question if it is possible to identify a cohort among non-frail patients that do not exhibit excess mortality as a result of suffering a hip fracture. According to 2018 data from Statistics Sweden, the Swedish Government agency responsible for producing official statistics, the 30-day mortality rate for citizens (calculated as the 1-year mortality rate multiplied by $\frac{30}{365}$) was between 0.1 and 0.2% for those between 65 and 79 years old [28]. Despite 50% of non-frail patients being within this range, the whole cohort still exhibited a significantly higher 30 days mortality rate at 1.2%. This could be attributable the higher comorbidity burden compared to the general population; however, it may also be a consequence of the significant stress that is put upon the body due to the initial trauma associated with the fracture as well as the subsequent trauma caused by the surgical intervention.

Both the fracture and pursuant surgery result in an activation of the sympathetic nervous system through the release or epinephrine, norepinephrine, and cortisol. This in turn induces a hyperadrenergic state which encompasses a cascade of reactions. These include the release of proinflammatory cytokines from the immune system as well as the induction of a catabolic state mediated by several endocrinological pathways, which may negatively impact recovery and wound healing [29–32]. If this state endures long enough, multiple organ systems may suffer additional insult, particularly the cardiovascular system [30, 31, 33–35]. This could explain why mortality due to cardiovascular events and multiorgan failure are the most common causes of mortality and that the highest incidences of mortality are observed during the first 30 days, both in the current investigation and previous studies [36–39].

Owing to the large degree of heterogeneity in hip fracture patients, it is vital to tailor care based on patients’ fitness for surgery, comorbidity burden, and degree of frailty. Beta-blocker therapy, hypothesized to mitigate the hyperadrenergic response, has recently been associated with lower mortality rates in hip fracture patients [40, 41]. The association also persists in patients with dementia [42], who are by and large frail [16], and appears to be larger in patients with an elevated cardiac risk [43]. Accordingly, beta-blockade may have a particular role to play in frail hip fracture patients as well. Another potential tool is the use of orthogeriatric care models to manage this vulnerable population. While the exact structure varies between institutions, multiple systematic reviews have found that orthogeriatric care is a cost-effective measure for reducing mortality in hip fracture patients [44–47]. Orthogeriatric care has also been associated with a lower risk of developing post-operative delirium [44], which has been strongly linked to elevated mortality and is particularly prevalent among frail patients [48, 49]. On the other hand, more traditional approaches to mitigating the postoperative mortality rate in hip fracture patients such as modifying the anesthetic and surgical technique as well as timing of surgery have failed to provide meaningful improvements in patient outcomes [50–53]. Likely, consequential progress will necessitate modifying the entire clinical care pathway for hip fracture patients [54]. However, it may also be worth considering if surgical management always is the optimal strategy, particularly in the most frail patients with a limited life expectancy [55].

This study consists of a national cohort of consecutively registered patients from the Swedish National Quality Registry for Hip Fracture Patients. This database is contributed to by the majority of orthopedic departments in Sweden with a high case coverage [56]. With the goal of contributing to equal and high quality care for hip fracture patients across the nation, Rikshoft serves as base for many hospitals’ quality assurance programs [22]. The merging of this dataset with the Swedish National Board of Health and Welfare Patient and Cause of Death registers allowed for a range of potential confounders to adjusted for in the analysis and helped avoid any loss to follow-up. Nevertheless, as this is a retrospective cohort study, there are limitations that bear mentioning. As this is a national cohort, the risk of selection bias is mitigated, yet the risk of residual confounding remains owing to unobserved or unknown variables. For example, data pertaining to anesthesia and all medications administered were not available in the current dataset. Furthermore, in order to ease the interpretation of the results and mirror clinical practice, patients were categorized as non-frail, pre-frail, and frail according to their OFS. However, in reality frailty exists on a spectrum with varying degrees of physical, cognitive, and psychosocial impairment [57, 58]. The risk of human errors when registering data must also be considered, particularly when recording the cause of death, which can be difficult to determine even in the best of circumstances. The probability that non-differential misclassification significantly affected the results is nonetheless low, given that the results of the current analyses are consistent with previous research. Finally, the analyses were limited to the outcomes available in the current dataset; consequently, the role frailty plays in postoperative pain, complications, and functional outcomes could not be included.

## Conclusion

Frailty is associated with a significantly increased risk of all-cause and cause-specific mortality at 30 and 90 days postoperatively. Across both timepoints, cardiovascular and respiratory events along with multiorgan failure were the most prevalent causes of mortality. Future studies are required to determine how to best optimize care for this vulnerable patient population.

## References

[1] Joseph B, Pandit V, Sadoun M, Zangbar B, Fain MJ, Friese RS (2014). Frailty in surgery. J Trauma Acute Care Surg.

[2] Clegg A, Young J, Iliffe S, Rikkert MO, Rockwood K (2013). Frailty in elderly people. Lancet Lond Engl.

[3] Robinson TN, Eiseman B, Wallace JI, Church SD, McFann KK, Pfister SM (2009). Redefining geriatric preoperative assessment using frailty, disability and co-morbidity. Ann Surg.

[4] Fried LP, Tangen CM, Walston J, Newman AB, Hirsch C, Gottdiener J (2001). Frailty in older adults: evidence for a phenotype. J Gerontol A Biol Sci Med Sci.

[5] Topinková E (2008). Aging, disability and frailty. Ann Nutr Metab.

[6] Joseph B, Zangbar B, Pandit V, Fain M, Mohler MJ, Kulvatunyou N (2016). Emergency general surgery in the elderly: too old or too frail?. J Am Coll Surg.

[7] Murphy PB, Savage SA, Zarzaur BL (2020). Impact of patient frailty on morbidity and mortality after common emergency general surgery operations. J Surg Res.

[8] Song Y, Wu Z, Huo H, Zhao P (2022). The impact of frailty on adverse outcomes in geriatric hip fracture patients: a systematic review and meta-analysis. Front Public Health.

[9] Ma Y, Wang A, Lou Y, Peng D, Jiang Z, Xia T (2022). Effects of frailty on outcomes following surgery among patients with hip fractures: a systematic review and meta-analysis. Front Med.

[10] Xu BY, Yan S, Low LL, Vasanwala FF, Low SG (2019). Predictors of poor functional outcomes and mortality in patients with hip fracture: a systematic review. BMC Musculoskelet Disord.

[11] Kim Y-P, Choe Y-R, Park J-H, Kim S, Won C-W, Hwang H-S (2019). Frailty index associated with all-cause mortality, long-term institutionalization, and hip fracture. Eur Geriatr Med.

[12] Kwak MJ, Digbeu BD, des Bordes J, Rianon N (2022). The association of frailty with clinical and economic outcomes among hospitalized older adults with hip fracture surgery. Osteoporos Int..

[13] Forssten MP, Cao Y, Trivedi DJ, Ekestubbe L, Borg T, Bass GA (2022). Developing and validating a scoring system for measuring frailty in patients with hip fracture: a novel model for predicting short-term postoperative mortality. Trauma Surg Acute Care Open BMJ Specialist J.

[14] Kanis JA, Odén A, McCloskey EV, Johansson H, Wahl DA, Cooper C (2012). A systematic review of hip fracture incidence and probability of fracture worldwide. Osteoporos Int J Establ Result Coop Eur Found Osteoporos Natl Osteoporos Found USA.

[15] Dhanwal DK, Dennison EM, Harvey NC, Cooper C (2011). Epidemiology of hip fracture: worldwide geographic variation. Indian J Orthop.

[16] Forssten MP, Ioannidis I, Mohammad Ismail A, Bass GA, Borg T, Cao Y (2022). Dementia is a surrogate for frailty in hip fracture mortality prediction. Eur J Trauma Emerg Surg [Internet].

[17] Nichols E, Steinmetz JD, Vollset SE, Fukutaki K, Chalek J, Abd-Allah F (2022). Estimation of the global prevalence of dementia in 2019 and forecasted prevalence in 2050: an analysis for the Global Burden of Disease Study 2019. Lancet Public Health Elsevier.

[18] Limb M (2016). World will lack 18 million health workers by 2030 without adequate investment, warns UN. BMJ Br Med J Publishing Group.

[19] AAMC Report Reinforces Mounting Physician Shortage [Internet]. AAMC. [Cited 10 Aug 2022]. Available from: https://www.aamc.org/news-insights/press-releases/aamc-report-reinforces-mounting-physician-shortage

[20] Projecting Hospitals’ Profit Margins Under Several Illustrative Scenarios: Working Paper 2016-04 | Congressional Budget Office [Internet]. [cited 10 Aug 2022]. Available from: https://www.cbo.gov/publication/51919

[21] Fact Sheet: Underpayment by Medicare and Medicaid | AHA [Internet]. [cited 10 Aug 2022]. Available from: https://www.aha.org/fact-sheets/2020-01-07-fact-sheet-underpayment-medicare-and-medicaid

[22] Om oss - Rikshöft [Internet]. RIKSHÖFT. [cited 3 Aug 2022]. Available from: https://www.xn--rikshft-e1a.se/om-oss

[23] WMA - The World Medical Association-WMA Declaration of Helsinki – Ethical Principles for Medical Research Involving Human Subjects [Internet]. [cited 13 May 2020]. Available from: https://www.wma.net/policies-post/wma-declaration-of-helsinki-ethical-principles-for-medical-research-involving-human-subjects/

[24] R Development Core Team. R: A Language and Environment for Statistical Computing [Internet]. Vienna, Austria: R Foundation for Statistical Computing; 2008. Available from: http://www.R-project.org/. Accessed 10 Aug 2022

[25] van de Ree CLP, Landers MJF, Kruithof N, de Munter L, Slaets JPJ, Gosens T (2019). Effect of frailty on quality of life in elderly patients after hip fracture: a longitudinal study. BMJ Open Br Med J Publishing Group.

[26] Orces CH (2016). Hip fracture-related mortality among older adults in the united states: analysis of the CDC WONDER multiple cause of death data. Epidemiol Res Int Hindawi.

[27] Chatterton BD, Moores TS, Ahmad S, Cattell A, Roberts PJ (2015). Cause of death and factors associated with early in-hospital mortality after hip fracture. Bone Jt J Br Editorial Soc Bone Jt Surg.

[28] Statistics Sweden. Mortality rate per 1,000 of the mean population by age and sex. Year 2000 - 2021 [Internet]. Stat. Database. [cited 4 Aug 2022]. Available from: http://www.statistikdatabasen.scb.se/pxweb/en/ssd/START__BE__BE0101__BE0101I/Dodstal/

[29] Loftus TJ, Efron PA, Moldawer LL, Mohr AM (2016). β-blockade Use for Traumatic Injuries and Immunomodulation: a review of proposed mechanisms and clinical evidence. Shock Augusta Ga.

[30] Dąbrowska AM, Słotwiński R (2014). The immune response to surgery and infection. Cent-Eur J Immunol.

[31] Moor D, Aggarwal G, Quiney N (2017). Systemic response to surgery. Surg Oxf Int Ed Elsevier.

[32] Desborough JP (2000). The stress response to trauma and surgery. Br J Anaesth.

[33] Lindenauer PK, Pekow P, Wang K, Mamidi DK, Gutierrez B, Benjamin EM (2005). Perioperative beta-blocker therapy and mortality after major noncardiac surgery. N Engl J Med.

[34] Cruickshank J, Degaute J, Kuurne T, Vincent J, Neil-Dwyer G, Hayes Y (1987). Reduction of stress/ catecholamine-induced cardiac necrosis by beta1-selective blockade. Lancet Elsevier.

[35] Maling HM, Highman B (1958). Exaggerated ventricular arrhythmias and myocardial fatty changes after large doses of norepinephrine and epinephrine in unanesthetized dogs. Am J Physiol-Leg Content Am Physiol Soc.

[36] Mohseni S, Joseph B, Peden CJ (2022). Mitigating the stress response to improve outcomes for older patients undergoing emergency surgery with the addition of beta-adrenergic blockade. Eur J Trauma Emerg Surg.

[37] Forssten MP, Ismail AM, Borg T, Ahl R, Wretenberg P, Cao Y (2021). Postoperative mortality in hip fracture patients stratified by the Revised Cardiac Risk Index: a Swedish nationwide retrospective cohort study. Trauma Surg Acute Care Open BMJ Specialist J.

[38] Mohammad Ismail A, Ahl R, Forssten MP, Cao Y, Wretenberg P, Borg T (2021). Beta-blocker therapy is associated with increased 1-year survival after hip fracture surgery: a retrospective cohort study. Anesth Analg.

[39] Ioannidis I, Mohammad Ismail A, Forssten MP, Ahl R, Cao Y, Borg T (2022). Surgical management of displaced femoral neck fractures in patients with dementia: a comparison in mortality between hemiarthroplasty and pins/screws. Eur J Trauma Emerg Surg.

[40] Ismail AM, Borg T, Sjolin G, Pourlotfi A, Holm S, Cao Y (2020). β-adrenergic blockade is associated with a reduced risk of 90-day mortality after surgery for hip fractures. Trauma Surg Acute Care Open BMJ Specialist J.

[41] Ahl R, Mohammad Ismail A, Borg T, Sjölin G, Forssten MP, Cao Y (2022). A nationwide observational cohort study of the relationship between beta-blockade and survival after hip fracture surgery. Eur J Trauma Emerg Surg.

[42] Ioannidis I, Mohammad Ismail A, Forssten MP, Cao Y, Bass GA, Borg T (2022). β-Adrenergic blockade in patients with dementia and hip fracture is associated with decreased postoperative mortality. Eur J Trauma Emerg Surg.

[43] Mohammad Ismail A, Ahl R, Forssten MP, Cao Y, Wretenberg P, Borg T (2022). The interaction between pre-admission β-blocker therapy, the revised cardiac risk index, and mortality in geriatric hip fracture patients. J Trauma Acute Care Surg.

[44] Van Heghe A, Mordant G, Dupont J, Dejaeger M, Laurent MR, Gielen E (2022). Effects of orthogeriatric care models on outcomes of hip fracture patients: a systematic review and meta-analysis. Calcif Tissue Int.

[45] Moyet J, Deschasse G, Marquant B, Mertl P, Bloch F (2019). Which is the optimal orthogeriatric care model to prevent mortality of elderly subjects post hip fractures? A systematic review and meta-analysis based on current clinical practice. Int Orthop.

[46] Grigoryan KV, Javedan H, Rudolph JL (2014). Orthogeriatric care models and outcomes in hip fracture patients: a systematic review and meta-analysis. J Orthop Trauma.

[47] Sabharwal S, Wilson H (2015). Orthogeriatrics in the management of frail older patients with a fragility fracture. Osteoporos Int.

[48] Tawab Saljuqi A, Hanna K, Asmar S, Tang A, Zeeshan M, Gries L (2020). Prospective evaluation of delirium in geriatric patients undergoing emergency general surgery. J Am Coll Surg.

[49] Kang SY, Seo SW, Kim JY (2019). Comprehensive risk factor evaluation of postoperative delirium following major surgery: clinical data warehouse analysis. Neurol Sci.

[50] Neuman MD, Feng R, Carson JL, Gaskins LJ, Dillane D, Sessler DI (2021). Spinal anesthesia or general anesthesia for hip surgery in older adults. N Engl J Med.

[51] Bhandari M, HEALTH Investigators (2019). Total hip arthroplasty or hemiarthroplasty for hip fracture. N Engl J Med..

[52] Borges FK, Bhandari M, Guerra-Farfan E, Patel A, Sigamani A, Umer M (2020). Accelerated surgery versus standard care in hip fracture (HIP ATTACK): an international, randomised, controlled trial. Lancet.

[53] Mohammad Ismail A, Forssten MP, Bass GA, Trivedi DJ, Ekestubbe L, Ioannidis I (2022). Mode of anesthesia is not associated with outcomes following emergency hip fracture surgery: a population-level cohort study. Trauma Surg Acute Care Open.

[54] Rogmark C (2020). Further refinement of surgery will not necessarily improve outcome after hip fracture. Acta Orthop.

[55] Loggers SAI, Willems HC, Van Balen R, Gosens T, Polinder S, Ponsen KJ (2022). Evaluation of quality of life after nonoperative or operative management of proximal femoral fractures in frail institutionalized patients: the FRAIL-HIP study. JAMA Surg.

[56] Rikshoft Arsrapport 2020 [Internet]. Lund, Sweden: Rikshoft; Available from: https://04e8d8b0-c67b-4aa0-a7e7-d272a37c2285.filesusr.com/ugd/3ac01b_2ce2105ebca7495c800465396dadaf89.pdf. Accessed 10 Aug 2022

[57] Crow RS, Lohman MC, Titus AJ, Bruce ML, Mackenzie TA, Bartels SJ (2018). Mortality risk along the frailty spectrum: data from the national health and nutrition examination survey 1999 to 2004. J Am Geriatr Soc.

[58] Jayanama K, Theou O, Blodgett JM, Cahill L, Rockwood K (2018). Frailty, nutrition-related parameters, and mortality across the adult age spectrum. BMC Med.

